# Plasma Vitamin E and the Risk of First Stroke in Hypertensive Patients: A Nested Case-Control Study

**DOI:** 10.3389/fnut.2021.734580

**Published:** 2021-11-03

**Authors:** Yun Song, Jingyi Li, Lishun Liu, Richard Xu, Ziyi Zhou, Benjamin Xu, Tengfei Lin, Ping Chen, Huan Li, Youbao Li, Chengzhang Liu, Xiao Huang, Binyan Wang, Yan Zhang, Jianping Li, Yong Huo, Fazheng Ren, Xiping Xu, Hao Zhang, Xianhui Qin

**Affiliations:** ^1^Key Laboratory of Precision Nutrition and Food Quality, Department of Nutrition and Health, College of Food Sciences and Nutritional Engineering, China Agricultural University, Beijing, China; ^2^Institute of Biomedicine, Anhui Medical University, Hefei, China; ^3^State Key Laboratory of Natural Medicines, Research Center of Biostatistics and Computational Pharmacy, China Pharmaceutical University, Nanjing, China; ^4^Tsinghua Shenzhen International Graduate School, Tsinghua University, Shenzhen, China; ^5^Department of Biostatistics, Johns Hopkins Bloomberg School of Public Health, Baltimore, MD, United States; ^6^Department of Epidemiology, Harvard T. H. Chan School of Public Health, Boston, MA, United States; ^7^College of Pharmacy, Jinan University, Guangzhou, China; ^8^National Clinical Research Study Center for Kidney Disease, Southern Medical University, Guangzhou, China; ^9^The State Key Laboratory for Organ Failure Research, Southern Medical University, Guangzhou, China; ^10^Renal Division, Nanfang Hospital, Southern Medical University, Guangzhou, China; ^11^Department of Scientific Research, Shenzhen Evergreen Medical Institute, Shenzhen, China; ^12^Department of Cardiology, Second Affiliated Hospital of Nanchang University, Nanchang, China; ^13^Department of Cardiology, Peking University First Hospital, Beijing, China

**Keywords:** vitamin E, first stroke, first ischemic stroke, sex, hypertensive males

## Abstract

**Background:** The association between plasma vitamin E levels and first stroke risk in men and women remains unclear.

**Objective:** We aimed to examine the prospective association between plasma vitamin E and first stroke, and evaluate the effect modifiers for the association, among hypertensive patients.

**Design:** The study sample was drawn from the China Stroke Primary Prevention Trial (CSPPT), which randomized a total of 20,702 hypertensive patients to a double-blind, daily treatment with either 10 mg enalapril and 0.8 mg folic acid or 10 mg enalapril alone. This nested case-control study, including 618 first stroke cases and 618 controls matched for age, sex, treatment group, and study site, was conducted after the completion of the CSPPT.

**Results:** The median follow-up duration was 4.5 years. Among men, a significantly higher risk of first stroke (adjusted OR, 1.67; 95%CI: 1.01, 2.77) was found for those with plasma vitamin E ≥7.1 μg/mL (≥quartile 1) compared with those with plasma vitamin E < 7.1 μg/mL. Subgroup analyses further showed that the association between vitamin E (≥7.1 vs. <7.1 μg/mL) and first stroke in men was significantly stronger in non-drinkers (adjusted OR, 2.64; 95%CI: 1.41, 4.96), compared to current drinkers (adjusted OR, 0.84; 95% CI: 0.43, 1.66, *P*-interaction = 0.008). However, there was no significant association between plasma vitamin E and first stroke in women (*P*-interaction between sex and plasma vitamin E = 0.048).

**Conclusions:** Among Chinese hypertensive patients, there was a statistically significant positive association between baseline plasma vitamin E and the risk of first stroke in men, but not in women.

**Clinical Trial Registration:** https://clinicaltrials.gov/ct2/show/NCT00794885, Identifier: NCT00794885.

## Introduction

Stroke is a leading cause of death in China and a major cause of long-term disability worldwide (1). Almost 76% of strokes are first attacks; accordingly, primary prevention is regarded as the best option to reduce the population burden of stroke (2). While many factors, including hypertension, diabetes mellitus, smoking, atrial fibrillation, hyperlipidemia, hyperhomocysteinemia, and alcohol intake have been identified as significant modifiable risk factors of stroke (3), there remains an urgent need to identify additional modifiable risk factors to further lower residual risk of first stroke and to develop safe, inexpensive, and effective primary prevention strategies to halt or reverse the rapidly rising trend of stroke in China and many parts of the world.

Vitamin E is an essential fat-soluble vitamin and is known for its antioxidant property. It is postulated that vitamin E protects against atherogenesis by acting as a scavenger of free radicals with subsequent reduced oxidation of low-density lipoprotein cholesterol, and by enacting several other favorable effects on plaque stability, platelet aggregation, and tendency to thrombosize (4–7). However, results from previous clinical trials of vitamin E supplementation (8, 9) and prospective studies of vitamin E intake (10) have been conflicting, ranging from a protective association, no clear association, to being a risk factor of cardiovascular disease (CVD), in particular, stroke.

A recent meta-analysis of randomized controlled trials reported that vitamin E supplementation had no significant effect on total, fatal or non-fatal stroke, but may offer some benefits in the prevention of ischemic stroke (11). However, prior randomized trials were mostly done in developed countries (11), and have mainly examined the effects of relatively high dose vitamin E supplementation in patients with CVD and/or diabetes, and did not consider the possible modifying effect of baseline plasma levels of vitamin E (12). Moreover, although dietary intake is known to correlate poorly with circulating vitamin levels, particularly vitamins C and E, most of the previous vitamin E studies were based on estimates of dietary vitamin E intake (10, 13, 14) rather than objective biomarker measurements. To date, only a few previous studies had been conducted to evaluate the prospective association between plasma vitamin E and the risk of stroke mortality (15) or ischemic stroke (16), and reported inconsistent findings. At the same time, few previous researches have comprehensively investigated the modifiers in the association between vitamin E and the risk of stroke. As such, the prospective association between circulating vitamin E and first stroke in population without the use of vitamin E supplements remains uncertain.

Furthermore, the issue has not been well-examined in populations with hypertension. This subgroup of the population deserves more attention, not only because it is less studied, but also because it is a subpopulation with a high risk for stroke. To address these significant gaps in knowledge outlined above, we aimed to explore the prospective association between baseline plasma vitamin E levels and the risk of first stroke, and evaluate whether the association can be modified by known risk factors of stroke, among hypertensive patients. We used a nested case-control study design leveraging the unique strength and resources of China Stroke Primary Prevention Trial (CSPPT) (17), which has high quality baseline and follow-up data and archived biospecimens.

## Methods

The parent study (the CSPPT) and the current study were approved by the Ethics Committee of the Institute of Biomedicine, Anhui Medical University, Hefei, China (FWA assurance number: FWA00001263). All participants gave prior written informed consent. The data that support the findings of this study will be available from the corresponding authors upon request, after the request is submitted and formally reviewed and approved by the Ethics Committee of the Institute of Biomedicine, Anhui Medical University.

### Study Design and Participants

The methods and major results of the CSPPT trial have been reported elsewhere (17–21). Briefly, the CSPPT was a multi-community, randomized, double-blind, controlled trial (17) conducted from May 19, 2008 to August 24, 2013 in 32 communities in Anqing, Anhui province and Lianyungang, Jiangsu province of China. Eligible participants were men and women aged 45–75 years with hypertension, defined as having a seated, resting systolic blood pressure (SBP) ≥140 mmHg or diastolic blood pressure (DBP) ≥90 mmHg at both the screening and recruitment visit or taking antihypertensive medication. The major exclusion criteria included history of physician-diagnosed stroke, myocardial infarction (MI), heart failure, post-coronary revascularization, and/or congenital heart disease.

In the CSPPT, a total of 20,702 eligible participants were randomly assigned, in a 1:1 ratio, to one of two treatment groups: a daily oral dose of one tablet containing 10 mg enalapril and 0.8 mg folic acid (the enalapril-folic acid group), or a daily oral dose of one tablet containing only 10 mg enalapril (the enalapril only group). Participants were followed up every 3 months. Over a median treatment duration of 4.5 years, a total of 637 incident stroke cases occurred in the CSPPT.

**Figure 1:**
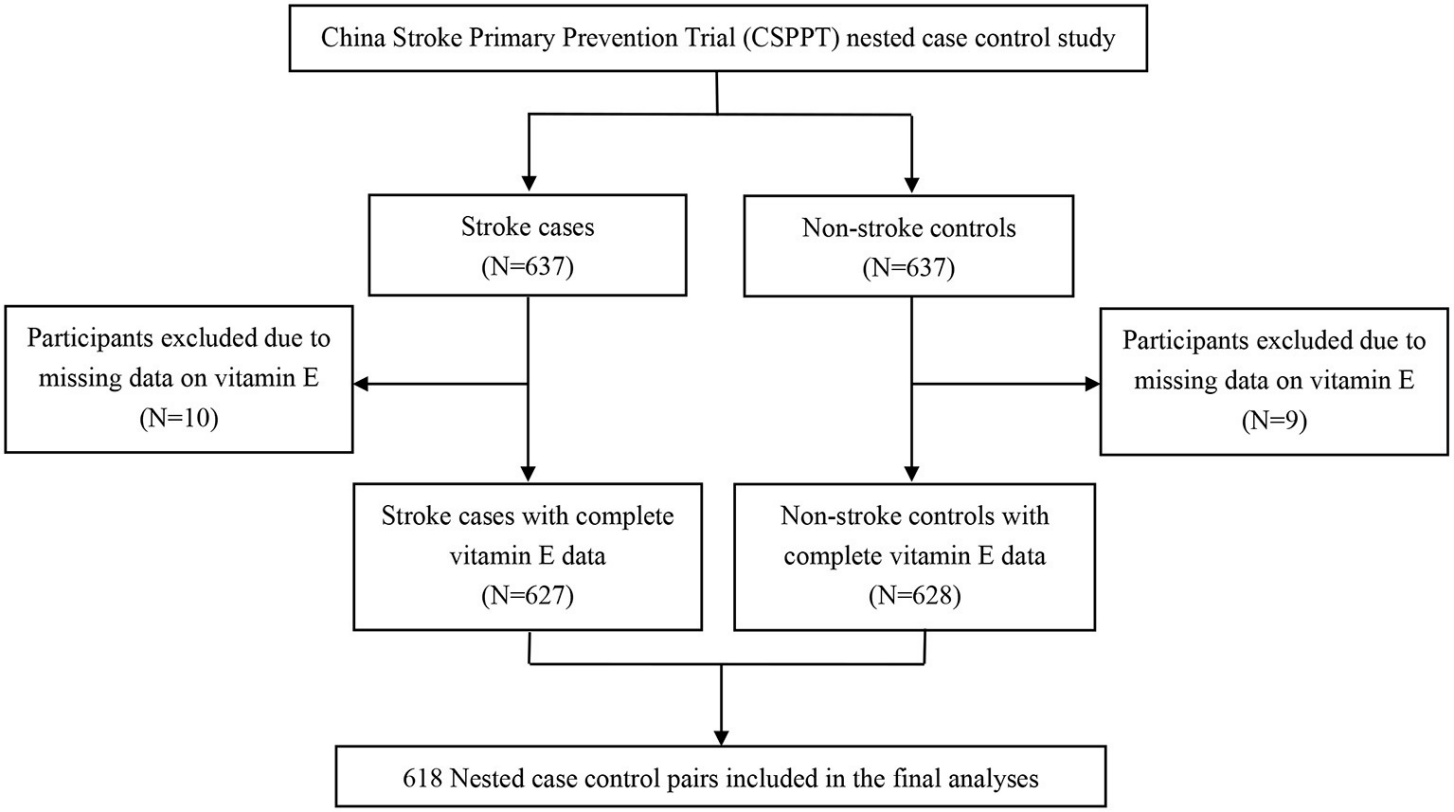
Flow chart of the study participants: A nested case control design based on the China Stroke Primary Prevention Trial (CSPPT).

Using data from the CSPPT, we conducted a nested case-control study. We identified all the participants from the CSPPT baseline participants who did not develop stroke during the follow-up as eligible controls. We then randomly matched eligible controls with first stroke cases by age (±1 year), sex, treatment group, and study sites in a 1:1 ratio. That is, the cumulative-incidence' sampling scheme was used. The initial sample consisted of 637 incident cases and 637 matched controls. Of the 1,274 selected cases or controls, 10 cases and 9 controls had missing values of vitamin E; as a result, 19 case/control pairs were removed either because of missing values of vitamin E or because of unpaired cases or controls. The final analyses included 618 matched case-control pairs (Figure 1).

### Assessment of Covariates

Baseline data collection was conducted by trained research staff according to a standard operating procedure. Each participant was interviewed using a standardized questionnaire designed specifically for this study. Current smoking was defined as having smoked at least 1 cigarette per day or ≥18 packs in the last year. Current drinking was defined as drinking alcohol at least 2 times per week in the last year.

Anthropometric measurements, including height, weight, and waist circumference, were taken using a standard operating procedure. Height was measured without shoes to the nearest 0.1 cm on a portable stadiometer. Weight was measured in light indoor clothing without shoes to the nearest 0.1 kg. Body mass index (BMI) was calculated as weight (kilograms)/height (meters) squared.

Seated BP was measured by trained research staff using a mercury manometer after the participants had rested for 10 min, following the standard method and with appropriately sized cuffs. Triplicate measurements on the same arm were taken, with at least 2 min between readings. The average of the three independent measures was used in the analyses.

### Outcomes Assessment

As detailed in an earlier report, the primary outcome was a first non-fatal or fatal stroke (ischemic or hemorrhagic), excluding subarachnoid hemorrhage and silent stroke. Secondary outcomes included first ischemic stroke and first hemorrhagic stroke. All the study outcomes were reviewed and adjudicated according to standard criteria by an independent Endpoint Adjudication Committee (17).

### Laboratory Assays

Overnight fasting venous blood samples were obtained from each study participant at baseline. Serum folate and vitamin B_12_ were measured by a commercial laboratory using a chemiluminescent immunoassay (New Industrial). Serum creatinine, total homocysteine (tHcy), fasting lipids, and fasting glucose were measured using automatic clinical analyzers (Beckman Coulter) at the core laboratory of the National Clinical Research Center for Kidney Disease, Nanfang Hospital, Guangzhou, China. Plasma vitamin E (alpha-tocopherol), vitamin D_3_ and retinol were measured by liquid chromatography with tandem quadrupole mass spectrometers (LC-MS/MS) in a commercial lab (Beijing DIAN Medical Laboratory, China). Plasma selenium, magnesium, copper, and zinc concentrations were measured by inductively coupled plasma mass spectrometry (ICP-MS) using Thermo Fisher iCAP Q ICP-MS in a commercial lab (Beijing DIAN Medical Laboratory, China).

### Statistical Analysis

Baseline characteristics are presented as means ± SDs for continuous variables and proportions for categorical variables. Differences in baseline characteristics between cases and controls by sex were compared using conditional logistic regression for categorical variables and generalized paired *t*-tests for continuous variables.

Variables that are known as traditional or suspected risk factors for stroke suggested in previous related guidelines (22) were chosen as the covariates in our current study. Odds ratios (ORs) of first stroke and its subtypes were estimated by modeling plasma vitamin E as quartiles (<quartile 1, quartile 1- < quartile 2, quartile 2- < quartile 3, and ≥quartile 3) and as categories (< quartile 1 vs. ≥ quartile 1) using conditional logistic regression, without and with adjustment for BMI (continuous), current smoking (yes vs. no), current alcohol drinking (yes vs. no), systolic blood pressure (SBP) at baseline (continuous), fasting blood glucose (continuous), total cholesterol (TC) (continuous), triglycerides (TG) (continuous), high-density lipoprotein cholesterol (HDL-C) (continuous), total homocysteine (tHcy) (continuous), folate (continuous), and estimated glomerular filtration rate (eGFR) (continuous) at baseline. In addition, possible modifications of the association between vitamin E (< quartile 1 vs. ≥quartile 1) and first stroke (total and ischemic) for males were assessed for the following variables: age (<65 vs. ≥65 years) (23), treatment group (enalapril vs. enalapril-folic acid), BMI (<24.1 (median) vs. ≥24.1 kg/m^2^), baseline SBP (<160 vs. ≥160 mmHg), current smoking status (no, yes), current alcohol drinking status (no, yes), TC (<5.2 vs. ≥5.2 mmol/L), tHcy [ <14.6 (median) vs. ≥14.6 μmol/L], and fasting glucose (<6.1 vs. ≥6.1 mmol/L or diabetes) at baseline. Potential interactions were examined by including the interaction terms into the logistic regression models. Of note, ORs of first hemorrhagic stroke in males were estimated using unconditional logistic regression due to relatively few hemorrhagic stroke cases, and all the above variables plus age, treatment groups, and study sites were adjusted.

A 2-tailed *P* < 0.05 was considered to be statistically significant. R software (version 3.4.3, www.R-project.org) and Empower (R) (version 2.17.8, www. empowerstats.com, X&Y Solutions, Inc. Boston, MA) were used for all statistical analyses.

## Results

### Study Participants and Characteristics

As illustrated in the flowchart, 618 stroke cases (497 ischemic stroke, 119 hemorrhagic stroke, and 2 unspecified types) and 618 matched controls (294 male pairs and 324 female pairs) with plasma vitamin E measurements were included in the current analysis (Figure 1). Comparisons of baseline characteristics of controls included in the current analysis and those not included among participants without first stroke during the treatment period, showed no obvious differences, and are presented in Supplementary Table 1. These results suggest that despite of matching with stroke cases, the selected controls for this report were similar to the overall non-stroke patients in the CSPPT.

Overall, mean ± SD values of vitamin E concentrations were higher in women (10.0 ± 3.5 μg/mL) than in men (9.4 ± 3.6 μg/mL; *P* < 0.001). Table 1 presents the characteristics of first stroke cases and controls by sex. Male and female stroke cases both had higher fasting glucose, baseline BP and time-averaged BP. Female stroke cases also had higher TC levels and fewer non-smokers.

**Table 1:** Characteristics of stroke cases and control subjects by sex^*^.

**Characteristics**	**Males**			**Females**		
	**Controls (*n* = 294)**	**Cases (*n* = 294)**	***P*** **-value**	**Controls (*n* = 324)**	**Cases (*n* = 324)**	***P*** **-value**
Vitamin E, μg/mL	9.2 (3.6)	9.6 (3.5)	0.131	10.0 (3.5)	10.0 (3.5)	0.886
Age, years	63 (7.1)	63 (7.1)	0.487	61.5 (7.3)	61.5 (7.3)	0.775
Body mass index, kg/m^2^	24.1 (3.1)	24.4 (3.5)	0.137	25.4 (3.6)	25.9 (4.0)	0.087
Waist-hip ratio	0.9 (0.1)	0.9 (0.1)	0.418	0.9 (0.1)	0.9 (0.1)	0.055
Current smoking, No. (%)	151 (51.4)	167 (56.8)	0.182	6 (1.9)	18 (5.6)	0.012
Current alcohol drinking, No. (%)	137 (46.6)	148 (50.3)	0.354	12 (3.7)	12 (3.7)	1.000
Enalapril-folic acid, No. (%)	119 (40.5)	119 (40.5)	1.000	153 (47.2)	153 (47.2)	1.000
Antihypertensive drugs, No. (%)	127 (43.2)	146 (49.7)	0.119	157 (48.5)	175 (54.0)	0.162
Blood pressure, mmHg						
Baseline SBP	166.3 (20.4)	176.2 (21.6)	<0.001	169.5 (19.8)	177.5 (23.9)	<0.001
Baseline DBP	94.9 (12.4)	98.2 (14.0)	0.001	92.3 (11.5)	96.8 (13.1)	<0.001
Time averaged SBP	138.9 (10.7)	148.5 (15.7)	<0.001	140 (11.4)	149.5 (15.3)	<0.001
Time averaged DBP	82.8 (7.9)	87.2 (10.6)	<0.001	81.5 (6.6)	86.3 (9.1)	<0.001
Laboratory results						
Total cholesterol, mmol/L	5.5 (1.1)	5.6 (1.3)	0.112	5.7 (1.2)	5.9 (1.2)	0.010
Triglycerides, mmol/L	1.5 (0.9)	1.5 (0.8)	0.982	1.7 (1.1)	1.8 (1.0)	0.646
HDL cholesterol, mmol/L	1.4 (0.4)	1.4 (0.4)	0.502	1.3 (0.3)	1.3 (0.3)	0.660
Total homocysteine, μmol/L	17.7 (10.6)	18.7 (12.2)	0.315	12.6 (4.4)	13.2 (4.9)	0.198
Fasting glucose, mmol/L	5.8 (1.4)	6.1 (2.2)	0.029	6.0 (1.9)	6.4 (2.6)	0.042
eGFR, ml/min/1.73 m^2^	90.8 (13.0)	89.3 (14.1)	0.231	92.5 (13.1)	92.4 (13.3)	0.980
Vitamin B_12_, pg/mL	428 (182.7)	403.5 (142.5)	0.071	409.5 (138.7)	421.7 (179.9)	0.294
Folate, ng/mL	7.8 (4.2)	7.3 (3.3)	0.075	8.8 (3.6)	8.6 (3.7)	0.353
Zinc, μg/dL	109.6 (30.5)	109.5 (37.5)	0.953	110.5 (26.5)	114.1 (47.8)	0.195
Copper, μg/dL	97.4 (20.2)	98.8 (19.0)	0.249	109.0 (20.4)	111.9 (20.6)	0.052
Retinol, μg/dL	80.1 (29.2)	77.5 (28.4)	0.252	64.5 (20.4)	62.9 (20.0)	0.319
Selenium, μg/dL	8.6 (2.0)	8.5 (2.1)	0.615	8.4 (2.1)	8.2 (1.9)	0.176
Magnesium, mg/L	20.3 (2.2)	20.3 (2.3)	0.716	20.5 (2.3)	20.6 (2.6)	0.547
Vitamin D_3_, ng/mL	21.4 (8.5)	21.0 (7.6)	0.474	17.6 (7.0)	17.6 (7.0)	0.881

* *For continuous variables, values are presented as mean (SD)*.

*DBP, diastolic blood pressure; SBP, systolic blood pressure; HDL, high density lipoprotein; eGFR, estimated glomerular filtration rate*.

Additional characteristics for male and female participants by vitamin E quartiles are presented in Supplementary Tables 2, 3. For male patients, higher plasma vitamin E concentrations were positively associated with TC, HDL-C, vitamin B_12_, folate, retinol, and vitamin D_3_ concentrations at baseline (Supplementary Table 2). Similar associations were found in female patients (Supplementary Table 3).

### Association Between Vitamin E and First Stroke

The median treatment duration was 4.5 years (IQR, 4.2–4.6 years). As shown in Table 2, when plasma vitamin E was assessed as quartiles in males, compared with <quartile 1 (<7.1 μg/mL), the adjusted ORs (95% CI) were 1.47 (0.83, 2.58), 1.88 (1.03, 3.45), and 1.93 (0.97, 3.84), respectively, for quartile 1- < quartile 2, quartile 2- < quartile 3, and ≥quartile 4 (*P* for trend = 0.038). Participants in ≥quartile 1 had a significantly higher risk of first stroke (OR, 1.67; 95% CI: 1.01, 2.77) compared with those in <quartile 1 (<7.1 μg/mL). Similar trends were found for first ischemic stroke and first hemorrhagic stroke.

**Table 2:** Relationship of plasma vitamin E with the risk of first stroke in males.

**Vitamin E, μg/mL**	**Cases/controls**	**Unadjusted**		Adjusted^*^	
		**OR (95% CI)**	***P-*** **value**	**OR (95% CI)**	***P-*** **value**
First stroke					
Per SD increment	294/294	1.16 (0.96, 1.40)	0.134	1.19 (0.93, 1.54)	0.166
Quartiles					
Q1 (<7.1)	66/81	*Ref*.		*Ref*.	
Q2 (7.1– <8.9)	69/78	1.12 (0.71, 1.77)	0.630	1.47 (0.83, 2.58)	0.186
Q3 (8.9– <11.3)	80/67	1.56 (0.95, 2.55)	0.077	1.88 (1.03, 3.45)	0.040
Q4 (≥11.3)	79/68	1.57 (0.93, 2.67)	0.092	1.93 (0.97, 3.84)	0.060
*P* for trend			0.040		0.038
Categories					
<7.1	66/81	*Ref*.		*Ref*.	
≥7.1	228/213	1.35 (0.91, 2.00)	0.137	1.67 (1.01, 2.77)	0.046
Ischemic stroke					
Per SD increment	244/244	1.21 (0.98, 1.50)	0.079	1.27 (0.94, 1.69)	0.115
Quartiles					
Q1 (<7.1)	54/68	*Ref*.		*Ref*.	
Q2 (7.1– <8.8)	59/63	1.24 (0.75, 2.05)	0.411	1.61 (0.86, 3.00)	0.137
Q3 (8.8– <11.2)	62/60	1.38 (0.81, 2.35)	0.238	1.54 (0.79, 2.99)	0.202
Q4 (≥11.2)	69/53	1.94 (1.07, 3.51)	0.028	2.39 (1.10, 5.18)	0.028
*P* for trend			0.029		0.046
Categories					
<7.1	54/68	*Ref*.		*Ref*.	
≥7.1	190/176	1.41 (0.91, 2.19)	0.124	1.67 (0.95, 2.93)	0.075
Hemorrhagic stroke				
Per SD increment	49/49	0.99 (0.66, 1.47)	0.948	1.07 (0.57, 2.01)	0.822
Quartiles					
Q1 (<7.7)	12/13	*Ref*.		*Ref*.	
Q2 (7.7– <9.4)	11/13	0.92 (0.30, 2.82)	0.879	1.74 (0.28, 10.83)	0.552
Q3 (9.4– <12.3)	15/9	1.81 (0.58, 5.64)	0.309	5.24 (0.82, 33.62)	0.081
Q4 (≥12.3)	11/14	0.85 (0.28, 2.59)	0.777	1.29 (0.22, 7.42)	0.777
*P* for trend			0.929		0.549
Categories					
<7.7	12/13	*Ref*.		*Ref*.	
≥7.7	37/36	1.11 (0.45, 2.76)	0.817	2.11 (0.47, 9.53)	0.332

* *Adjusted for body mass index (BMI), smoking status, alcohol drinking, systolic blood pressure (SBP) at baseline, fasting blood glucose, total cholesterol (TC), triglycerides (TG), high-density lipoprotein cholesterol (HDL-C), total homocysteine (tHcy), folate, as well as estimated glomerular filtration rate (eGFR) at baseline*.

Among male participants, stroke cases and controls had no difference in concomitant medication use during the treatment period (Supplementary Table 4). Moreover, during the treatment period, there was no difference in concomitant medication use among vitamin E quartile concentrations in male participants (Supplementary Table 5). As expected, further adjustments for the use of medications during the treatment period (Supplementary Table 6) did not substantially alter the findings. Moreover, further adjustments for baseline serum vitamin B_12_, and plasma zinc, copper, retinol, vitamin D_3_, selenium, and magnesium (Supplementary Table 7); further adjustments for baseline educational levels and marital status (Supplementary Table 8); or categorizing vitamin E according to quartiles in the total population (Supplementary Table 9), also did not materially change the results.

Nevertheless, no clear association of plasma vitamin E with first stroke or its subtypes was found in females (Table 3). Sex significantly modified the association between plasma vitamin E (<quartile 1 vs. ≥quartile 1) with first stroke (*P*-interaction = 0.048).

**Table 3:** Relationship of plasma vitamin E with the risk of first stroke in females^*^.

**Vitamin E, μg/mL**	**Cases/controls**	**Unadjusted**		**Adjusted**	
		**OR (95% CI)**	***P-*** **value**	**OR (95% CI)**	***P-*** **value**
First stroke					
Per SD increment	324/324	1.01 (0.84, 1.22)	0.886	0.92 (0.73, 1.15)	0.455
Quartiles					
Q1 (<7.6)	80/82	*Ref*.		*Ref*.	
Q2 (7.6– <9.5)	75/87	0.90 (0.57, 1.42)	0.662	0.84 (0.51, 1.40)	0.504
Q3 (9.5– <12.0)	93/69	1.39 (0.86, 2.24)	0.181	1.11 (0.63, 1.95)	0.716
Q4 (≥12.0)	76/86	0.91 (0.54, 1.52)	0.718	0.64 (0.34, 1.20)	0.165
*P* for trend			0.803		0.322
Categories					
<7.6	80/82	*Ref*.		*Ref*.	
≥7.6	244/242	1.04 (0.70, 1.56)	0.838	0.88 (0.55, 1.40)	0.591
Ischemic stroke					
Per SD increment	254/254	1.04 (0.84, 1.28)	0.716	0.91 (0.70, 1.19)	0.487
Quartiles					
Q1 (<7.6)	62/65	*Ref*.		*Ref*.	
Q2 (7.6– <9.6)	54/73	0.82 (0.49, 1.37)	0.449	0.81 (0.44, 1.47)	0.485
Q3 (9.6– <11.9)	76/51	1.59 (0.93, 2.73)	0.093	1.32 (0.68, 2.56)	0.405
Q4 (≥11.9)	62/65	1.08 (0.60, 1.95)	0.797	0.75 (0.35, 1.57)	0.440
*P* for trend			0.300		0.795
Categories					
<7.6	62/65	*Ref*.		*Ref*.	
≥7.6	192/189	1.08 (0.69, 1.70)	0.729	0.94 (0.54, 1.62)	0.815
Hemorrhagic stroke				
Per SD increment	69/69	0.90 (0.61, 1.34)	0.610	1.10 (0.64, 1.87)	0.738
Quartiles					
Q1 (<7.7)	17/18	*Ref*.		*Ref*.	
Q2 (7.7– <9.3)	20/14	1.51 (0.53, 4.33)	0.439	1.77 (0.47, 6.56)	0.396
Q3 (9.3– <12.2)	17/17	1.02 (0.34, 3.04)	0.975	1.35 (0.31, 5.85)	0.688
Q4 (≥12.2)	15/20	0.76 (0.25, 2.31)	0.625	0.96 (0.22, 4.19)	0.955
*P* for trend			0.430		0.734
Categories					
<7.7	17/18	*Ref*.		*Ref*.	
≥7.7	52/51	1.11 (0.45, 2.73)	0.819	1.43 (0.43, 4.74)	0.557

* *Adjusted for body mass index (BMI), smoking status, alcohol drinking, systolic blood pressure (SBP) at baseline, fasting blood glucose, total cholesterol (TC), triglycerides (TG), high-density lipoprotein cholesterol (HDL-C), total homocysteine (tHcy), folate, as well as estimated glomerular filtration rate (eGFR) at baseline*.

### Subgroup Analyses

**Figure 2:**
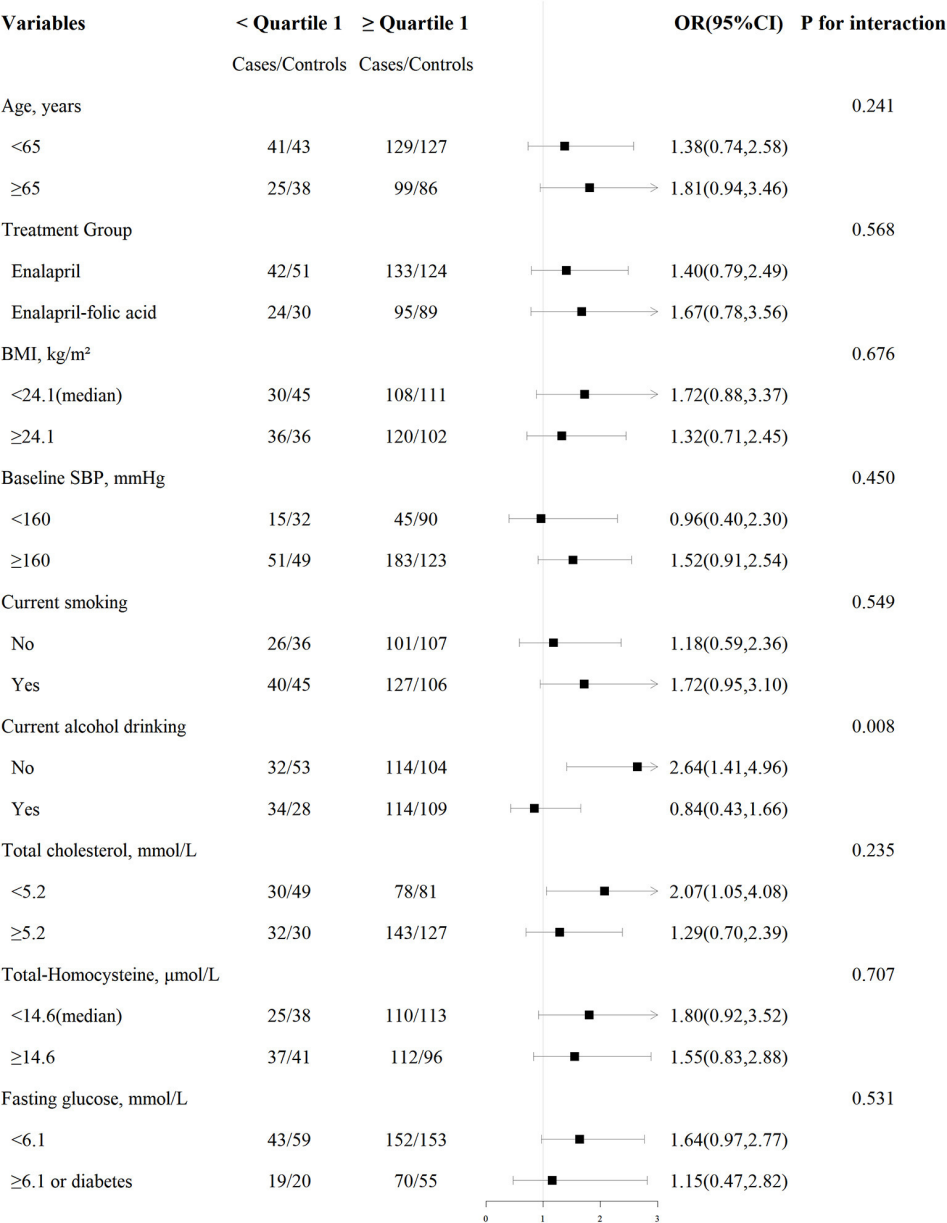
Association between plasma vitamin E (< quartile 1 vs. ≥quartile 1) and risk of first total stroke among males in various subgroups. Each subgroup analysis adjusted, if not stratified, for age, treatment group, study site, body mass index (BMI), smoking status, alcohol drinking, systolic blood pressure at baseline (SBP), fasting blood glucose, total cholesterol (TC), triglycerides (TG), high-density lipoprotein cholesterol (HDL-C), total homocysteine (tHcy), folate, as well as estimated glomerular filtration rate (eGFR) at baseline.

Among males, stratified analyses were performed to further assess the association between plasma vitamin E (<quartile 1 vs. ≥quartile 1) and the risk of first stroke in various subgroups (Figure 2, Supplementary Table 10). A stronger positive association between vitamin E and first stroke was observed in non-drinkers (adjusted OR, 2.64; 95% CI: 1.41, 4.96) compared to current drinkers (adjusted OR, 0.84; 95% CI: 0.43, 1.66, *P*-interaction = 0.008).

None of the other variables in males, including age, treatment group, body mass index, baseline SBP, time-averaged SBP, smoking status, total cholesterol, total homocysteine, eGFR, TG, HDL-C, fasting glucose, folate, retinol levels, copper levels, vitamin B_12_ levels, zinc levels, magnesium levels, selenium levels, and vitamin D_3_ levels, showed significant effect modifications on the association between plasma vitamin E and the risk of first stroke (*P*-interaction > 0.05 for all these stratified variables).

## Discussion

In this nested case-control study derived from the CSPPT, using objective measure of plasma vitamin E (alpha-tocopherol) levels, we demonstrated a significant positive association between plasma vitamin E and subsequent risk of first stroke in male hypertensive patients. Furthermore, our subgroup analyses demonstrated that the positive relation of plasma vitamin E with first stroke was more pronounced in male participants who were non-drinkers.

Previous studies have examined the relation of blood vitamin E with mortality risk in non-Chinese population, but the results have been inconclusive (24, 25). Buijsse et al. (24) found that plasma concentrations of α-tocopherol were not associated with all-cause or cause-specific mortality in elderly subjects who participated in a European prospective study. Wright et al. (25) showed that higher serum α-tocopherol levels were related to moderately lower total and cause-specific mortality in older male smokers. However, only a few previous studies (15, 16) have examined the association between blood vitamin E concentrations and the risk of stroke. Nagao et al. (15) found that serum α-tocopherol level was not associated with any type of cardiovascular death in Japanese men; however, in women, it was inversely associated with hemorrhagic stroke mortality. Of note, this study (15) did not have data about incident stroke. At the same time, Hak et al. (16) reported that levels of α-tocopherol were not associated with ischemic stroke in the Physicians' Health Study, in which about 35% of the participants used multiple vitamins supplements. More importantly, only a part of the participants had hypertension in the two previous studies, and both of the studies did not account for some important potential confounders, such as eGFR, other nutrients, and mean BP over the follow-up period. Therefore, the findings from the above studies suggested that the association between circulating vitamin E and first stroke in hypertensive population without vitamin E supplements remains uncertain.

Our study offered a chance to evaluate the dose-response relation of plasma vitamin E with first stroke in hypertensive patients by leveraging the well-established CSPPT, a randomized controlled trial. Our study has contributed to several new insights. First, in hypertensive men, there was a significantly positive relationship of plasma vitamin E with incident risk of first stroke. The potential mechanism by which higher vitamin E may increase risk of stroke is still unclear, but it is biologically plausible. In 1981, Roberts had reported that high dosages of vitamin E were associated with thrombophlebitis, hypertension, fatigue, and increased levels of free cholesterol in low-density, and very-low-density lipoproteins (26). In fact, a series of previous studies found that alpha-tocopherol (alpha-TOH) can act as a pro-oxidant for isolated low-density lipoprotein (LDL) (27–29), by reaction of the α-tocopheroxyl radical with polyunsaturated fatty acid moieties in the lipid (30).

Second, this study suggested a possible sex difference. We did not find a significant relationship of vitamin E with first stroke in females. Two possible explanations for this include: (1) estrogen acts to prevent oxidative damage of the endothelium and oxidation of LDL in the atherosclerotic process, which begins in childhood and continues into adulthood (31–33); (2) the low incidence of first stroke in women may have weakened the statistical power of the current study. Overall, the exact mechanisms underlying the sex difference in the association between plasma vitamin E levels and the risk of first stroke remain to be understood.

Third, we found that among males, alcohol drinking was a significant effect modifier. A stronger association between vitamin E and first stroke was found in non-drinkers. The EPIC-CVD case-cohort study found that alcohol intake was positively associated with the risk of different stroke subtypes (34). Consistently, in the prospective China Kadoorie Biobank, genotype-predicted mean alcohol intake had a continuously positive log-linear association with the risk of intracerebral hemorrhage and ischaemic stroke (35). Mechanisms linking alcohol drinking and first stroke risk remain incompletely understood; however, available evidence suggests that alcohol drinking may induce oxidative stress and vascular injury (36–38). Consistently, alpha-tocopherol (alpha-TOH) has also been suggested as a pro-oxidant (21). Of note, in our current study, the lowest first stroke risk was found in non-drinking males with low vitamin E levels, and similar first stroke risks were observed in current drinking males and/or those with high vitamin E levels. Therefore, we speculate that alcohol drinking and high vitamin E levels may share some common pathway in the development of first stroke. As such, the detrimental effects of alcohol drinking may attenuate the positive relation of plasma vitamin E and first stroke. However, more research is needed to validate our findings, and explore its underlying mechanisms.

## Strengths and Limitations

Our study had high quality baseline and follow-up epidemiological and clinical data including validation of incident stroke cases, and a comprehensive analysis of many important risk factors or confounders of stroke. However, there are also several limitations of the current study. First, although the current study's mean ± SD vitamin E level (9.4 ± 3.6 μg/mL in male and 10.0 ± 3.5 μg/mL in female) seemed to be comparable with other nested case-control studies (31–34), the generalizability of our findings to other populations with different demographic and clinical characteristics requires caution. Second, our analyses are based on a single baseline measurement of vitamin E and therefore may not reflect levels over a longer period. Third, our study only measured α-tocopherol in plasma and could therefore not specify the levels of other different chemical forms of vitamin E (e.g., γ-tocopherol and tocotrienol). Fourth, we did not have detailed dietary information, and could not adjusted quality of diet in the regress models. Finally, there is an inherit limitation of any *post-hoc* analysis; we cannot exclude the possibility of residual confounding despite our control of important epidemiologic and clinical covariates in the analyses. Additional studies are required to confirm our results.

## Conclusions

In Chinese hypertensive patients, higher baseline plasma vitamin E was significantly associated with an increased risk of first stroke in males. Our findings, if further confirmed, may inform clinical and nutritional guidelines on the primary prevention of stroke, by including plasma vitamin E as a potentially modifiable risk factor, especially in hypertensive males. However, more studies are needed to verify our results and to further examine the biological mechanisms underlying the associations.

## Data Availability Statement

The raw data supporting the conclusions of this article will be made available by the authors, without undue reservation.

## Ethics Statement

The studies involving human participants were reviewed and approved by Ethics Committee of the Institute of Biomedicine, Anhui Medical University, Hefei, China. The patients/participants provided their written informed consent to participate in this study.

## Author Contributions

XX, XQ, YS, LL, and HZ: study concept and design. XQ, YS, JinL, LL, HZ, BX, TL, ZZ, PC, YL, BW, JiaL, YZ, CL, YH, and FR: conduct of study. YS, JinL, LL, and TL: data collection and analysis. XQ, YS, JinL, LL, and RX: drafting of the manuscript. XQ, YS, JinL, LL, HZ, ZZ, PC, YL, XH, BW, JL, YZ, CL, YH, and FR: critical review and revision of the manuscript for important intellectual content. All authors contributed to the article and approved the submitted version.

## Funding

The study was supported by the National Key Research and Development Program (2016YFE0205400, 2018ZX09739010, and 2018ZX09301034003), the Department of Science and Technology of Guangdong Province (2020B121202010), the Science and Technology Planning Project of Guangzhou, China (201707020010), the Science, Technology and Innovation Committee of Shenzhen (GJHS20170314114526143, JSGG20180703155802047), and the Economic, Trade and Information Commission of Shenzhen Municipality (20170505161556110, 20170505160926390, and 201705051617070).

## Conflict of Interest

The authors declare that the research was conducted in the absence of any commercial or financial relationships that could be construed as a potential conflict of interest.

## Publisher's Note

All claims expressed in this article are solely those of the authors and do not necessarily represent those of their affiliated organizations, or those of the publisher, the editors and the reviewers. Any product that may be evaluated in this article, or claim that may be made by its manufacturer, is not guaranteed or endorsed by the publisher.
