# Supplementary material for: Plasma Vitamin E and the Risk of First Stroke in Hypertensive Patients: A Nested Case-Control Study
Source: Front Nutr. 2021 Nov 3;8:734580. doi: 10.3389/fnut.2021.734580 (PMC8595403; doi:10.3389/fnut.2021.734580)
Supplement: Supplementary file 1 [file Table_1.docx]

**Online Supplemental Material**

**Title:** Plasma vitamin E and the risk of first stroke in hypertensive patients: a nested case-control study

**Short title:** Plasma vitamin E and first stroke

**Correspondence:**

Hao Zhang, Ph.D.

Beijing Advanced Innovation Center for Food Nutrition and Human Health, College of Food Science and Nutritional Engineering, China Agricultural University, Beijing, China

Email: zhanghaocau@cau.edu.cn;

Phone: 86-010-62738589

Fax: 86-010-62736344

or

Xianhui Qin, M.D.

National Clinical Research Study Center for Kidney Disease; the State Key Laboratory for Organ Failure Research; Renal Division, Nanfang Hospital, Southern Medical University, Guangzhou, China

Email: pharmaqin@126.com

Phone: 86-20-61641591

Fax: 86-20-87281713

**Please send the proofs to Dr. Xianhui Qin,** [**pharmaqin@126.com**](mailto:pharmaqin@126.com)**.**

**Supplemental Table 1.** Baseline characteristics of controls included in the current analysis and those not included among participants without first stroke during the treatment period.

**Supplemental Table 2.** Population characteristics stratified by vitamin E quartiles for males.

**Supplemental Table 3.** Population characteristics stratified by Vitamin E quartiles for females.

**Supplemental Table 4.** Concomitant medication usage during the treatment period for stroke cases and controls.

**Supplemental Table 5.** Concomitant medication usage during the treatment period by quartiles of Vitamin E.

**Supplemental Table 6.** Association between plasma vitamin E and risk of first stroke in males with further adjustment for concomitant medication usage during the treatment period.

**Supplemental Table 7.** Association between plasma vitamin E and risk of first stroke in males with further adjustments for baseline serum vitamin B12, and plasma zinc, copper, retinol, vitamin D_3_, selenium, and magnesium.

**Supplemental Table 8.** Association between plasma vitamin E and risk of first stroke in males with further adjustments for baseline educational levels and marital status.

**Supplemental Table 9.** Association of vitamin E, categorized according to quartiles in the total population, with first stroke in males and females

**Supplemental Table 10.** Association between plasma vitamin E (<7.1 vs. ≥7.1μg/mL) and risk of first stroke among males in additional various subgroups.

**Supplemental Table 1. Baseline characteristics of controls included in the current analysis and those not included among participants without first stroke during the treatment period*.**

| Characteristics | Selected Controls (*n*=618) | Excluded (*n*=19447) |
| --- | --- | --- |
| Body mass index, kg/m^2^ | 24.8 (3.4) | 24.9 (3.7) |
| Waist-hip ratio | 0.9 (0.1) | 0.9 (0.1) |
| Current smoking, No. (%) |  |  |
| No | 461 (74.6) | 14915 (76.7) |
| Yes | 157 (25.4) | 4524 (23.3) |
| Current alcohol drinking, No. (%) |  |  |
| No | 469 (75.9) | 14790 (76.1) |
| Yes | 149 (24.1) | 4646 (23.9) |
| Enalapril-folic acid, No. (%) | 272 (44.0) | 9794 (50.4) |
| Antihypertensive drugs, No. (%) | 284 (46.0) | 8925 (45.9) |
| **Study site** |  |  |
| Anqing | 114 (18.4) | 4985 (25.6) |
| Lianyungang | 504 (81.6) | 14462 (74.4) |
| **Blood pressure, mmHg** |  |  |
| Baseline SBP | 168.0 (20.1) | 166.5 (20.2) |
| Baseline DBP | 93.5 (12) | 94 .0(11.9) |
| Time averaged SBP during the  treatment period | 139.5 (11.1) | 139.5 (11.2) |
| Time averaged DBP during the  treatment period | 82.1 (7.3) | 83.0 (7.6) |
| **Laboratory results** |  |  |
| Total cholesterol, mmol/L | 5.6 (1.2) | 5.5 (1.2) |
| Triglycerides, mmol/L | 1.6 (1.0) | 1.7 (1.2) |
| HDL cholesterol, mmol/L | 1.4 (0.4) | 1.3 (0.4) |
| Total homocysteine, μmol/L | 15.0 (8.3) | 14.4 (8.3) |
| Fasting glucose, mmol/L | 5.9 (1.7) | 5.8 (1.7) |
| eGFR, ml/min/1.73m^2^ | 91.7 (13.1) | 93.6 (13.2) |
| Vitamin B_12_, pg/mL | 418.2 (161.2) | 412.2 (156.4) |
| Folate, ng/mL | 8.3 (3.9) | 8.6 (3.9) |

*****Abbreviations: DBP, diastolic blood pressure; SBP, systolic blood pressure; HDL, high density lipoprotein; eGFR, estimated glomerular filtration rate.

For continuous variables, values are presented as mean (SD).

**Supplemental Table 2. Population characteristics stratified by vitamin E quartiles for males*.**

| Characteristics | Stratified by Vitamin E quartiles (μg/mL) | | | | *P value* |
| --- | --- | --- | --- | --- | --- |
|  | <7.1 (n=147) | 7.1-<8.9 (*n*=147) | 8.9-<11.3 (*n*=147) | ≥11.3 (n=147) |  |
| Vitamin E, μg/mL | 5.6 (1.2) | 7.9 (0.5) | 10.0 (0.7) | 14.2 (2.9) | < 0.001 |
| Age, year | 62.8 (7.7) | 63.6 (6.7) | 62.6 (7.2) | 62.9 (6.8) | 0.655 |
| Body mass index, kg/m^2^ | 24.2 (3.3) | 24.4 (3.2) | 24.4 (3.4) | 23.9 (3.3) | 0.586 |
| Waist-hip ratio | 0.9 (0.1) | 0.9 (0.1) | 0.9 (0.1) | 0.9 (0.1) | 0.453 |
| Current smoking, No. (%) | 85 (57.8) | 65 (44.2) | 77 (52.4) | 91 (61.9) | 0.016 |
| Current alcohol drinking, No. (%) | 62 (42.2) | 58 (39.5) | 76 (51.7) | 89 (60.5) | < 0.001 |
| Enalapril-folic acid, No. (%) | 54 (36.7) | 68 (46.3) | 55 (37.4) | 61 (41.5) | 0.317 |
| Antihypertensive drugs, No. (%) | 71 (48.3) | 61 (41.5) | 71 (48.3) | 70 (47.6) | 0.586 |
| Blood pressure, mmHg |  |  |  |  |  |
| Baseline SBP | 173.8 (24.2) | 168.6 (18.4) | 169.2 (22.5) | 173.4 (20.4) | 0.067 |
| Baseline DBP | 97.2 (14.4) | 95.3 (13.1) | 96.8 (12.4) | 97.0 (13.3) | 0.586 |
| Time averaged SBP | 144.6 (14.8) | 141.4 (14.3) | 143.9 (12.8) | 144.9 (14.9) | 0.138 |
| Time averaged DBP | 86.4 (10.1) | 83.4 (8.6) | 85.5 (10.1) | 84.7 (9.3) | 0.048 |
| Educational levels |  |  |  |  | 0.907 |
| Illiterate | 56 (38.1) | 56 (38.1) | 63 (42.9) | 65 (44.2) |  |
| Primary level | 38 (25.9) | 39 (26.5) | 33 (22.4) | 35 (23.8) |  |
| Elementary or higher levels | 53 (36.1) | 52 (35.4) | 51 (34.7) | 47 (32) |  |
| Marital status |  |  |  |  | 0.452 |
| Never married | 2 (1.4) | 0 (0.0) | 2 (1.4) | 2 (1.4) |  |
| Married | 119 (81.0) | 126 (86.3) | 127 (88.2) | 123 (84.8) |  |
| Divorced/widow/others | 26 (17.7) | 20 (13.7) | 15 (10.4) | 20 (13.8) |  |
| **Laboratory results** |  |  |  |  |  |
| Total cholesterol, mmol/L | 5.1 (1.1) | 5.2 (0.9) | 5.7 (1.2) | 6.2 (1.3) | < 0.001 |
| Triglycerides, mmol/L | 1.4 (0.7) | 1.4 (0.6) | 1.5 (1.0) | 1.6 (1.0) | 0.073 |
| HDL cholesterol, mmol/L | 1.2 (0.3) | 1.3 (0.3) | 1.4 (0.4) | 1.5 (0.5) | < 0.001 |
| Total homocysteine, μmol/L | 21.1 (13.8) | 18.2 (11.7) | 17 (10.0) | 16.6 (9.1) | 0.003 |
| Fasting glucose, mmol/L | 5.7 (1.4) | 6.0 (2.0) | 6.1 (2.1) | 5.9 (1.8) | 0.298 |
| eGFR, ml/min/1.73m^2^ | 88.6 (15.4) | 89.3 (11.6) | 90.6 (13.7) | 91.7 (13.2) | 0.220 |
| Vitamin B_12_, pg/mL | 386.8 (157.4) | 402.4 (146.7) | 419.8 (152.4) | 453.6 (190.5) | 0.004 |
| Folate, ng/mL | 6.5 (3.0) | 7.5 (3.3) | 8.0 (3.8) | 8.4 (4.6) | < 0.001 |
| Zinc, μg/dL | 109.6 (26.2) | 104.7 (31.2) | 108.0 (23.2) | 115.9 (49.1) | 0.041 |
| Copper, μg/dL | 101.8 (18.8) | 100.1 (19.2) | 97.8 (21.4) | 92.8 (17.9) | < 0.001 |
| Retinol, μg/dL | 67.4 (24.1) | 70.9 (23.3) | 79.6 (24.0) | 97.4 (33.1) | < 0.001 |
| Selenium, μg/dL | 8.5 (2.2) | 8.3 (1.9) | 8.7 (2.1) | 8.6 (2.0) | 0.446 |
| Magnesium, mg/L | 21.0 (2.1) | 20.4 (2.0) | 20.3 (2.2) | 19.6 (2.4) | < 0.001 |
| Vitamin D_3_, ng/mL | 19.4 (7.8) | 20.3 (7.5) | 21.7 (8.1) | 23.3 (8.2) | < 0.001 |

*****Abbreviations: DBP, diastolic blood pressure; SBP, systolic blood pressure; HDL, high density lipoprotein; eGFR, estimated glomerular filtration rate. For continuous variables, values are presented as mean (SD).

Differences in characteristics were compared using χ2 tests for categorical variables and ANOVA tests for continuous variables.

**Supplemental Table 3. Population characteristics stratified by Vitamin E quartiles for females*.**

| Characteristics | Stratified by Vitamin E quartiles (μg/mL) | | | | *P value* |
| --- | --- | --- | --- | --- | --- |
|  | <7.6 (n=162) | 7.6-<9.5 (*n*=162) | 9.5-<12.0 (*n*=162) | ≥12.0 (n=162) |  |
| Vitamin E, μg/mL | 6.1 (1.3) | 8.6 (0.6) | 10.7 (0.7) | 14.6 (2.7) | < 0.001 |
| Age, year | 60.1 (7.6) | 61.3 (7.4) | 61.7 (6.9) | 63.1 (7.1) | 0.002 |
| Body mass index, kg/m^2^ | 25.5 (3.7) | 25.5 (3.8) | 26.1 (4.1) | 25.5 (3.6) | 0.446 |
| Waist-hip ratio | 0.9 (0.1) | 0.9 (0.1) | 0.9 (0.1) | 0.9 (0.1) | 0.717 |
| Current smoking, No. (%) | 4 (2.5) | 1 (0.6) | 8 (4.9) | 11 (6.8) | 0.018 |
| Current alcohol drinking, No. (%) | 6 (3.7) | 5 (3.1) | 5 (3.1) | 8 (5.0) | 0.785 |
| Enalapril-folic acid, No. (%) | 70 (43.2) | 83 (51.2) | 68 (42.0) | 85 (52.5) | 0.129 |
| Antihypertensive drugs, No. (%) | 87 (53.7) | 77 (47.5) | 94 (58.0) | 74 (45.7) | 0.099 |
| Blood pressure, mmHg |  |  |  |  |  |
| Baseline SBP | 169.0 (20.8) | 173.6 (22.8) | 175.0 (21.7) | 176.4 (23.3) | 0.017 |
| Baseline DBP | 93.3 (11.6) | 95.2 (13.5) | 94.8 (12.4) | 94.8 (12.5) | 0.522 |
| Time averaged SBP | 143.4 (14.1) | 145.2 (15.3) | 146.4 (14.2) | 144.1 (13.6) | 0.252 |
| Time averaged SBP | 83.6 (7.9) | 85.0 (9.1) | 84.1 (8.4) | 82.9 (7.5) | 0.134 |
| Educational levels |  |  |  |  | 0.296 |
| Illiterate | 131 (80.9) | 145 (89.5) | 140 (86.4) | 140 (87) |  |
| Primary level | 20 (12.3) | 13 (8) | 12 (7.4) | 12 (7.5) |  |
| Elementary or higher levels | 11 (6.8) | 4 (2.5) | 10 (6.2) | 9 (5.6) |  |
| Marital status |  |  |  |  | NA |
| Never married | 0 (0.0) | 0 (0.0) | 0 (0.0) | 0 (0.0) |  |
| Married | 136 (84.0) | 128 (79.0) | 138 (86.2) | 135 (83.9) |  |
| Divorced/widow/others | 26 (16.0) | 34 (21.0) | 22 (13.8) | 26 (16.1) |  |
| Laboratory results |  |  |  |  |  |
| Total cholesterol, mmol/L | 5.2 (1.2) | 5.5 (0.9) | 5.9 (1.2) | 6.4 (1.2) | < 0.001 |
| Triglycerides, mmol/L | 1.5 (0.7) | 1.6 (0.8) | 1.8 (0.9) | 2.0 (1.5) | < 0.001 |
| HDL cholesterol, mmol/L | 1.2 (0.3) | 1.3 (0.3) | 1.3 (0.3) | 1.4 (0.4) | < 0.001 |
| Total homocysteine, μmol/L | 12.9 (4.4) | 13.0 (4.3) | 12.4 (4.6) | 13.3 (5.2) | 0.429 |
| Fasting glucose, mmol/L | 5.9 (1.7) | 5.9 (1.8) | 6.3 (2.1) | 6.7 (3.1) | 0.002 |
| eGFR, ml/min/1.73m^2^ | 91.9 (13.2) | 91.6 (12.5) | 94.5 (12.9) | 91.9 (14.0) | 0.180 |
| Vitamin B_12_, pg/mL | 404.5 (164.6) | 403.4 (138.4) | 419.1 (152.8) | 435.5 (182.9) | 0.238 |
| Folate, ng/mL | 8.0 (3.5) | 8.6 (3.4) | 8.8 (3.5) | 9.5 (4.0) | 0.004 |
| Zinc, μg/dL | 109.7 (24.2) | 109.8 (57.6) | 113.3 (30.6) | 116.6 (33.5) | 0.323 |
| Copper, μg/dL | 113.8 (20.7) | 111.8 (21.7) | 110.5 (19.3) | 105.8 (19.6) | 0.004 |
| Retinol, μg/dL | 52.1 (15.5) | 58.3 (14.6) | 66.2 (21.2) | 78.2 (18.8) | < 0.001 |
| Selenium, μg/dL | 8.3 (2.0) | 8.2 (1.8) | 8.5 (2.2) | 8.1 (2.0) | 0.372 |
| Magnesium, mg/L | 21.1 (2.6) | 20.7 (2.3) | 20.4 (2.3) | 20.0 (2.5) | < 0.001 |
| Vitamin D_3_, ng/mL | 15.9 (6.4) | 17.5 (6.9) | 17.6 (7.2) | 19.5 (7.3) | < 0.001 |

*****Abbreviations: SBP, systolic blood pressure; DBP, diastolic blood pressure; HDL, high-density lipoprotein; eGFR, estimated glomerular filtration rate.

For continuous variables, values are presented as mean (SD).

Differences in characteristics were compared using χ2 tests for categorical variables and ANOVA tests for continuous variables.

**Supplemental Table 4.** **Concomitant medication usage during the treatment period for stroke cases and controls*.**

| Medication | Controls | Cases | *P* value |
| --- | --- | --- | --- |
| **Males** |  |  |  |
| No. (%) | 294 | 294 |  |
| Anti-hypertension drugs |  |  |  |
| Calcium channel blockers | 241 (82) | 248 (84.4) | 0.419 |
| Diuretics | 157 (53.4) | 156 (53.1) | 0.929 |
| Glucose-lowering drugs | 4 (1.4) | 10 (3.4) | 0.076 |
| Antiplatelet drugs | 4 (1.4) | 6 (2.0) | 0.526 |
| Lipoprotein-lowering drugs | 0 (0.0) | 0 (0.0) | - |
| **Females** |  |  |  |
| No. (%) | 324 | 324 |  |
| Anti-hypertension drugs |  |  |  |
| Calcium channel blockers | 255 (78.7) | 274 (84.6) | 0.048 |
| Diuretics | 151 (46.6) | 189 (58.3) | 0.001 |
| Glucose-lowering drugs | 11 (3.4) | 15 (4.6) | 0.413 |
| Antiplatelet drugs | 1 (0.3) | 6 (1.9) | 0.047 |
| Lipoprotein-lowering drugs | 0 (0.0) | 4 (1.2) | 0.019 |

*****Differences in concomitant medication usage were compared using conditional logistic regression for categorical

variables and generalized paired *t* tests for continuous variables.

Regular concomitant medication usage was defined as 180 or more cumulative days of taking the drug of interest.

**Supplemental Table 5. Concomitant medication usage during the treatment period** **by quartiles of Vitamin E*.**

| Medication | Vitamin E, μg/mL | | | | *P* value |
| --- | --- | --- | --- | --- | --- |
| **Males** |  |  |  |  |  |
| Vitamin E quartiles (μg/mL) | Q1(<7.1) | Q2(7.1-<8.9) | Q3(8.9-<11.3) | Q4(≥11.3) |  |
| No. (%) | 147 | 147 | 147 | 147 |  |
| Anti-hypertension drugs |  |  |  |  |  |
| Calcium channel blockers | 125 (85.0) | 114 (77.6) | 130 (88.4) | 120 (81.6) | 0.077 |
| Diuretics | 88 (59.9) | 72 (49.0) | 75 (51.0) | 78 (53.1) | 0.266 |
| Glucose-lowering drugs | 1 (0.7) | 3 (2) | 4 (2.7) | 6 (4.1) | 0.306 |
| Antiplatelet drugs | 2 (1.4) | 1 (0.7) | 4 (2.7) | 3 (2.0) | 0.714 |
| Lipoprotein-lowering drugs | 0 (0.0) | 0 (0.0) | 0 (0.0) | 0 (0.0) | - |
| **Females** |  |  |  |  |  |
| Vitamin E quartiles (μg/mL) | Q1(<7.6) | Q2(7.6-<9.5) | Q3(9.5-<12.0) | Q4(≥12.0) |  |
| No. (%) | 162 | 162 | 162 | 162 |  |
| Anti-hypertension drugs |  |  |  |  |  |
| Calcium channel blockers | 129 (79.6) | 134 (82.7) | 134 (82.7) | 132 (81.5) | 0.876 |
| Diuretics | 87 (53.7) | 80 (49.4) | 91 (56.2) | 82 (50.6) | 0.608 |
| Glucose-lowering drugs | 2 (1.2) | 8 (4.9) | 6 (3.7) | 10 (6.2) | 0.132 |
| Antiplatelet drugs | 0 (0.0) | 5 (3.1) | 0 (0.0) | 2 (1.2) | 0.020 |
| Lipoprotein-lowering drugs | 0 (0.0) | 2 (1.2) | 1 (0.6) | 1 (0.6) | 0.905 |

*****Differences in concomitant medication usage were compared by using χ^2^ tests.

Regular concomitant medication usage was defined as 180 or more cumulative days of taking the drug of interest.

**Supplemental Table 6.** **Association between plasma vitamin E and risk of first stroke in males with further adjustments for concomitant medication usage during the treatment period*.**

| Vitamin E, μg/mL | Cases/Controls  (No.) | Adjusted | |
| --- | --- | --- | --- |
|  |  | OR (95% CI) | *P* value |
| First Stroke |  |  |  |
| Quartiles |  |  |  |
| Q1(<7.1) | 66/81 | *Ref.* |  |
| Q2(7.1-<8.9) | 69/78 | 1.48 (0.83,2.62) | 0.183 |
| Q3(8.9-<11.3) | 80/67 | 1.90 (1.03,3.49) | 0.039 |
| Q4(≥11.3) | 79/68 | 1.87 (0.93,3.76) | 0.077 |
| *P* for trend |  |  | 0.047 |
| Categories |  |  |  |
| Q1(<7.1) | 66/81 | *Ref.* |  |
| Q2-Q4(≥7.1) | 228/213 | 1.68 (1.01,2.80) | 0.047 |

*****Adjusted for body mass index (BMI), smoking status, alcohol drinking, systolic blood pressure (SBP) at baseline, fasting blood glucose, total cholesterol (TC), triglycerides (TG), high-density lipoprotein cholesterol (HDL-C), total homocysteine (tHcy), folate, estimated glomerular filtration rate (eGFR) at baseline, glucose lowering drug use, calcium channel blockers and diuretics use during the treatment period.

**Supplemental Table 7. Association between plasma vitamin E and risk of first stroke in males with further**

**adjustments for baseline serum vitamin B12, and plasma zinc, copper, retinol, vitamin D3, selenium,**

**and magnesium*.**

| Vitamin E, μg/mL | Cases/Controls | Adjusted | |
| --- | --- | --- | --- |
|  |  | OR (95% CI) | *P* value |
| Quartiles |  |  |  |
| Q1(<7.1) | 66/81 | *Ref.* |  |
| Q2(7.1-<8.9) | 69/78 | 1.66 (0.90,3.06) | 0.104 |
| Q3(8.9-<11.3) | 80/67 | 2.92 (1.45,5.86) | 0.003 |
| Q4(≥11.3) | 79/68 | 3.45 (1.59,7.48) | 0.002 |
| *P* for trend |  |  | <0.001 |
| Categories |  |  |  |
| Q1(<7.1) | 66/81 | *Ref.* |  |
| Q2-Q4(≥7.1) | 228/213 | 2.19 (1.25,3.84) | 0.006 |

*****Adjusted for body mass index (BMI), smoking status, alcohol drinking, systolic blood pressure (SBP) at baseline, fasting blood glucose, total cholesterol (TC), triglycerides (TG), high-density lipoprotein cholesterol (HDL-C), total homocysteine (tHcy), folate, estimated glomerular filtration rate (eGFR), vitamin B_12_, plasma zinc, copper, retinol, vitamin D3, selenium, and magnesium at baseline.

**Supplemental Table 8. Association between plasma vitamin E and risk of first stroke in males with further adjustments for baseline educational levels and marital status.**

| Vitamin E, μg/mL | Cases/Controls | Adjusted | |
| --- | --- | --- | --- |
|  |  | OR (95% CI) | *P* value |
| Quartiles |  |  |  |
| Q1(<7.1) | 66/81 | *Ref.* |  |
| Q2(7.1-<8.9) | 69/78 | 1.35 (0.76,2.39) | 0.309 |
| Q3(8.9-<11.3) | 80/67 | 1.71 (0.91,3.19) | 0.093 |
| Q4(≥11.3) | 79/68 | 1.84 (0.91,3.70) | 0.089 |
| *P* for trend |  |  | 0.060 |
| Categories |  |  |  |
| Q1(<7.1) | 66/81 | *Ref.* |  |
| Q2-Q4(≥7.1) | 228/213 | 1.54 (0.92,2.58) | 0.100 |

*****Adjusted for body mass index (BMI), smoking status, alcohol drinking, systolic blood pressure (SBP) at baseline, fasting blood glucose, total cholesterol (TC), triglycerides (TG), high-density lipoprotein cholesterol (HDL-C), total homocysteine (tHcy), folate, estimated glomerular filtration rate (eGFR), educational levels and marital status at baseline.

**Supplemental Table 9 Association of vitamin E, categorized according to quartiles in the total population, with first stroke in males and females *.**

| Vitamin E, μg/mL | Cases/Controls | Unadjusted | | Adjusted | |
| --- | --- | --- | --- | --- | --- |
|  |  | OR (95% CI) | *P* value | OR (95% CI) | *P* value |
| **Male** |  |  |  |  |  |
| Per SD increment | 294/294 | 1.16 (0.96,1.40) | 0.134 | 1.19 (0.93,1.54) | 0.166 |
| Quartiles |  |  |  |  |  |
| Q1 (<7.3) | 75/92 | *Ref.* |  | *Ref.* |  |
| Q2 (7.3-<9.3) | 74/78 | 1.19 (0.76,1.86) | 0.457 | 1.76 (0.99,3.13) | 0.054 |
| Q3 (9.3-<11.7) | 76/60 | 1.64 (1.01,2.66) | 0.044 | 2.04 (1.13,3.69) | 0.018 |
| Q4 (≥11.7) | 69/64 | 1.43 (0.84,2.41) | 0.184 | 1.74 (0.88,3.45) | 0.112 |
| *P* for trend |  |  | 0.077 |  | 0.069 |
| Categories |  |  |  |  |  |
| Q1 (<7.3) | 75/92 | *Ref.* |  | *Ref.* |  |
| Q2-4 (≥7.3) | 219/202 | 1.38 (0.94,2.02) | 0.102 | 1.86 (1.13,3.07) | 0.015 |
| **Female** | |  |  |  |  |
| Per SD increment | 324/324 | 1.01 (0.84,1.22) | 0.886 | 0.92 (0.73,1.15) | 0.455 |
| Quartiles |  |  |  |  |  |
| Q1 (<7.3) | 71/75 | *Ref.* |  | *Ref.* |  |
| Q2 (7.3-<9.3) | 74/80 | 1.00 (0.62,1.63) | 0.985 | 0.94 (0.54,1.61) | 0.810 |
| Q3 (9.3-<11.7) | 92/78 | 1.26 (0.77,2.06) | 0.358 | 1.01 (0.56,1.80) | 0.983 |
| Q4 (≥11.7) | 87/90 | 1.06 (0.63,1.79) | 0.827 | 0.77 (0.41,1.47) | 0.430 |
| *P* for trend |  |  | 0.642 |  | 0.495 |
| Categories |  |  |  |  |  |
| Q1 (<7.3) | 71/75 | *Ref.* |  | *Ref.* |  |
| Q2-4 (≥7.3) | 253/248 | 1.10 (0.72,1.69) | 0.663 | 0.93 (0.57,1.53) | 0.775 |

*****Each subgroup analysis adjusted, if not stratified, for age, treatment group, study site, body mass index (BMI), smoking status, alcohol drinking, systolic blood pressure at baseline (SBP), fasting blood glucose, total cholesterol (TC), triglycerides (TG), high-density lipoprotein cholesterol (HDL-C), total homocysteine (tHcy), folate, and estimated glomerular filtration rate (eGFR) at baseline.

**Supplemental Table 10. Association between plasma vitamin E [1st Quartile (reference) (Q1, <7.1μg/mL) *vs.* 2-4 Quartile (Q2-Q4, ≥7.1μg/mL)] and risk of first stroke among males in additional various subgroups *.**

| Subgroup variables | Q1 | Q2-4 | OR (95%CI) | *P for interaction* |
| --- | --- | --- | --- | --- |
|  | Cases/Controls | Cases/Controls |  |  |
| eGFR, ml/min/1.73m^2^ |  |  |  | 0.586 |
| <92.0 (median) | 37/38 | 111/99 | 1.40(0.76,2.57) |  |
| ≥92.0 | 25/41 | 110/109 | 1.69(0.86,3.32) |  |
| Triglycerides, mmol/L |  |  |  | 0.774 |
| <1.3 (median) | 35/44 | 101/107 | 1.60(0.84,3.05) |  |
| ≥1.3 | 31/36 | 124/104 | 1.50(0.79,2.85) |  |
| HDL cholesterol, mmol/L |  |  |  | 0.653 |
| <1.3 (median) | 39/52 | 106/91 | 1.38(0.75,2.52) |  |
| ≥1.3 | 27/28 | 120/120 | 1.38(0.69,2.73) |  |
| Folate, ng/ml |  |  |  | 0.641 |
| <6.9 (median) | 46/47 | 105/90 | 1.38(0.78,2.43) |  |
| ≥6.9 | 17/33 | 119/119 | 1.78(0.84,3.76) |  |
| Retinol, μg/dL |  |  |  | 0.545 |
| <74.5 (median) | 41/55 | 106/91 | 1.60(0.88,2.91) |  |
| ≥74.5 | 25/26 | 122/122 | 1.43(0.69,2.97) |  |
| Copper, μg/dL |  |  |  | 0.990 |
| <97.1 (median) | 25/32 | 118/115 | 1.89(0.91,3.92) |  |
| ≥97.1 | 41/47 | 109/94 | 1.52(0.84,2.77) |  |
| Vitamin B_12_, pg/mL |  |  |  | 0.767 |
| <379.7 (median) | 40/45 | 108/95 | 1.50(0.81,2.76) |  |
| ≥379.7 | 23/35 | 116/114 | 1.73(0.87,3.45) |  |
| Zinc, μg/dL |  |  |  | 0.527 |
| <103.5 (median) | 31/38 | 116/105 | 1.32(0.71,2.48) |  |
| ≥103.5 | 34/41 | 111/104 | 1.92(0.99,3.74) |  |
| Magnesium, mg/L |  |  |  | 0.642 |
| <20.4 (median) | 21/36 | 124/109 | 1.73(0.85,3.49) |  |
| ≥20.4 | 45/43 | 103/100 | 1.57(0.84,2.93) |  |
| Selenium, μg/dL |  |  |  | 0.159 |
| <8.3 (median) | 37/38 | 112/103 | 1.20(0.63,2.28) |  |
| ≥8.3 | 29/41 | 115/106 | 2.09(1.07,4.07) |  |
| Vitamin D_3_, ng/mL |  |  |  | 0.200 |
| <20.8 (median) | 41/44 | 108/101 | 1.15(0.64,2.07) |  |
| ≥20.8 | 25/37 | 120/112 | 2.40(1.16,4.99) |  |

*****Each subgroup analysis adjusted, if not stratified, for age, treatment group, study site, body mass index (BMI), smoking status, alcohol drinking, systolic blood pressure at baseline (SBP), fasting blood glucose, total cholesterol (TC), triglycerides (TG), high-density lipoprotein cholesterol (HDL-C), total homocysteine (tHcy), folate, and estimated glomerular filtration rate (eGFR) at baseline.
